# Apples and oranges: PTSD patients and healthy individuals are not comparable in their subjective and physiological responding to emotion induction and bilateral stimulation

**DOI:** 10.3389/fpsyg.2024.1406180

**Published:** 2024-06-12

**Authors:** Valeska Pape, Gebhard Sammer, Bernd Hanewald, Eva Schäflein, Fritz Rauschenbach, Markus Stingl

**Affiliations:** ^1^Center for Psychiatry and Psychotherapy Justus-Liebig-University Giessen, Giessen, Germany; ^2^Clinic and Policlinic for Psychiatry and Psychotherapy, University of Rostock, Rostock, Germany; ^3^Faculty of Medicine, Department of Psychotherapy and Psychosomatic Medicine, Technische Universität Dresden, Dresden, Germany

**Keywords:** bilateral stimulation, EMDR, emotion, physiology, PTSD, startle reflex, tactile stimulation

## Abstract

**Objectives:**

Bilateral stimulation is a core element of Eye Movement Desensitization and Reprocessing Therapy, a psychotherapeutic intervention for the treatment of Posttraumatic Stress Disorder (PTSD). Promising previous findings showed measurable physiological effects of bilateral stimulation in healthy individuals, but studies that replicated these findings in PTSD patients are sparse.

**Methods:**

23 patients with PTSD and 30 healthy controls were confronted with affective standard scripts (pleasant, neutral, unpleasant) while bilateral tactile stimulation was applied. Monolateral and no stimulation served as control conditions. Noise-induced startle reflex response (valence measure) and galvanic skin response (arousal measure) were used for physiological responses and the valence and arousal scale of the Self-Assessment-Manikin for subjective responses.

**Results:**

Both groups showed a subjective distress reduction for unpleasant scripts and a subjective attention increase for positive scripts under bilateral stimulation. In healthy individuals, this was also for physiological measures, and a general startle-reducing effect of bilateral stimulation in the absence of affective stimuli was found. In PTSD patients, however, the effects were restricted on the subjective level, and no concomitant physiological effects were observed.

**Conclusions and significance:**

The findings indicate, that generalizing the effects of BLS in healthy individuals to PTSD patients may be problematic. The herein-reported group differences can be explained by PTSD-specific peculiarities in emotion processing and cognitive processing style.

## Physiological characteristics of PTSD compared to healthy individuals

Posttraumatic stress disorder (PTSD) is an immediate or even delayed response to a traumatic event, characterized by frequent reliving of the trauma, avoidance of trauma-associated stimuli and physiological hyperarousal (Diagnostic and Statistical Manual of Mental Disorders DSM-V, Wittchen et al., 1997; APA, 2013). The disorder has a 1-year prevalence of 2.3 to 9.1% (Schein et al., 2021) and causes high socioeconomic costs due to reduced life quality, absenteeism and loss of productivity (Habetha et al., 2012).

PTSD is also associated with significant physiological changes. According to the trauma memory model, experiencing a traumatic event should lead to massive involuntary sympathetic activation and adrenaline release, which is physiologically reflected in an increase in heart rate (HR), skin conductivity (SC), and startle responsiveness. If there is a renewed confrontation with trauma-associated stimuli later, a comparable aversive reaction should be triggered, even if there is objectively no longer any danger, and an accompanied physiological hyper-arousal is postulated, even if there are no affective stimuli at all (e.g., Sartory et al., 2013; Brewin, 2018).

The following section outlines the group differences between PTSD patients and healthy individuals with respect to various physiological variables and response patterns.

### Group differences in physiological basis parameters

The assumption of group differences in physiological basis parameters has only been partially confirmed: Increased startle responsiveness and slower habituation to loud sounds in patients with PTSD could only be demonstrated if this was measured via HR, but not if measured via orbicularis oculi electromyography (EMG) and SC (Metzger et al., 1999; Orr et al., 2003), except from very stressful situation with electric shocks, which did increase the EMG-measured startle reflex (Morgan et al., 1995). Some authors found basally increased RR intervals in PTSD patients (Blanchard, 1990), while other authors did not (McFall et al., 1992). With regard to SC and frontalis EMG, *no* over-activity correlating with the severity of PTSD was found (McDonagh-Coyle et al., 2001; Rothbaum et al., 2001). These discrepancies might partially be explained by the different power of the studies (Prins et al., 1995).

### Group differences in the processing of trauma-associated vs. general affective stimuli

Other authors compared the emotional responses to affectively relevant images or scripts in subjects with vs. without PTSD. They found an exaggerated physiological reactivity to trauma-associated stimuli in PTSD patients. As physiological measures, the noise-induced startle reflex, SC (McNally et al., 1987; Pitman et al., 1990; Orr et al., 1993; McDonagh-Coyle et al., 2001; Tarrier et al., 2002; Pole, 2007), HR, blood pressure (BP), and frontalis EMG (Blanchard, 1990; Pitman et al., 1990; Orr et al., 1993; McDonagh-Coyle et al., 2001) were used. These measures were able to discriminate between PTSD subjects and healthy subjects (Orr et al., 1993), even when subjects were asked to alter/dissimulate their physiological responses (Gerardi et al., 1989, SC and HR). The processing of non-trauma-associated aversive stimuli, in contrast, was *not* different between the groups (Tarrier et al., 2002), but a significantly diminished emotional reactivity of pleasant and neutral stimuli in PTSD patients was found (McDonagh-Coyle et al., 2001).

### Group differences in responding to bilateral stimulation

In the last decades, Eye Movement Desensitization and Reprocessing (EMDR) has been introduced as an effective treatment for PTSD, expanding the understanding of trauma therapy techniques by considering mechanisms of action beyond mere habituation (Shapiro, 1989, 1996, 2002, 2017; Bisson et al., 2013). In contrast to classical prolonged exposure trauma therapy, in the imaginative exposure phase of EMDR, bilateral alternating sensory stimuli are presented, which may consist of induced eye movements (visual stimulation), rhythmic touching of the body surface (tactile stimulation on, e.g., the hands), or alternating sounds acoustic stimulation (Shapiro, 2017). According to the adaptive information processing model (AIP model, Shapiro, 2001; Solomon and Shapiro, 2008) this should improve the processing and integration of stressful memories and provide affective relief.

On the one hand, numerous randomized clinical trials exist comparing the effectivity of EMDR treatment in PTSD patients to other methods of trauma therapy (Van Etten and Taylor, 1998; Ironson et al., 2002; Power et al., 2002). According to meta-analyses, the PTSD symptom-reducing effect of EMDR is comparable to other forms of trauma-focusing treatments such as Trauma-focused Cognitive Behavioral Therapy (Tf-CBT) (e.g., Chen et al., 2014; Khan et al., 2018). On the other hand, there is an increasing number of laboratory studies examining the underlying working mechanisms of EMDR in experimental settings (see an overview by Houben et al., 2020). These dismantling studies do not focus on EMDR as a whole but on potential mechanisms of action such as the specific type of stimulation. Some of these studies addressed PTSD patients (Wilson et al., 1996; Servan-Schreiber et al., 2006; Elofsson et al., 2008), some of them healthy individuals (Andrade et al., 1997; Barrowcliff et al., 2003; Gunter and Bodner, 2008; Engelhard et al., 2010; Nieuwenhuis et al., 2013). Besides subjective distress reduction (Servan-Schreiber et al., 2006), diverse physiological effects of bilateral stimulation were found. In PTSD patients, for example, a stimulation-induced decrease in skin conductance and heart rate was observed (Elofsson et al., 2008). Investigations in healthy individuals also found a valence-dependent reduction in startle reflex potentiation (indicating a reduced distress) during imagination of negative scripts and an increase in SCR (indicating an attention increase) during imagination of positive scripts (Reichel et al., 2021). However, there have not been many reviews specifically addressing the differential effects of stimulation on the two groups,

In the only meta-analysis found for this topic, no significant differences between clinical populations and healthy individuals were found (Lee and Cuijpers, 2013). These findings, however, are limited: They were created in full EMDR sessions and do not examine the effects of specific components of EMDR such as bilateral stimulation (BLS). Furthermore, previous studies on the temporal stability of EMDR effects mainly refer to EMDR as a whole, reporting conflicting results (Wilson et al., 1997; Carlson et al., 1998; Devilly et al., 1998; Macklin et al., 2000; Marcus et al., 2004). Finally, no objective measurement parameters were regarded, i.e., the findings are restricted to subjective data which may be disturbed by social desirability effects. Consequently, a direct comparison of PTSD patients and healthy individuals in the same study design which should include both subjective and physiological measures as well as follow up testing is needed.

## Aims and hypotheses

The aim of the present research project is to expand the knowledge on subjective and physiological differences between PTSD patients and healthy individuals while emotional processing and their responsiveness to bilateral stimulation. For this purpose, an in *sensu* confrontation with imagination scripts of different valences (negative vs. neutral vs. positive) under different stimulation conditions (bilateral vs. monolateral vs. no stimulation) is to be carried out. During the imagination process, physiological and psychometric arousal parameters as well as physiological and psychometric valence parameters are to be measured.

In a first step, previous findings concerning group differences in physiological basis parameters and emotional reactivity should be replicated. For this question, comparisons are made between the groups (PTSD patients vs. control subjects) and between the different script categories (negative vs. neutral vs. positive vs. no script), whereby only trials without stimulation are considered.

In a second step, group differences in emotional reactivity under the different stimulation conditions (bilateral stimulation vs. monolateral stimulation vs. no stimulation) are focused. For this question, trials with bilateral stimulations are compared with those with monolateral and none stimulation.

The following hypotheses are tested:

*Hypotheses on general emotional reactivity*

*H1:* A significant interaction effect between the factors ‘script category’ (negative vs. neutral as main contrast) and ‘group’ (PTSD patients vs. control subjects) is expected, i.e., PTSD patients show (1) a stronger increase in aversive feelings and a stronger increase in arousal when imagining negative scripts (2) a lower increase in positive feelings and a lower increase in arousal when imagining positive scripts (compared to neutral scripts).

*Hypotheses on the effect of bilateral stimulation*

*H2:* A significant interaction effect between the factors ‘stimulation type’ (bilateral vs. no stimulation as main contrast) and ‘script category’ (negative vs. neutral) is expected, i.e., with bilateral stimulation the imagined affective (i.e., negative or positive) situations are experienced less intense (i.e., aversive or positive) and less arousal-generating than without stimulation (compared to neutral scripts). This applies equally to PTSD patients and healthy people, i.e., there is no difference between the groups.

## Materials and methods

### Participants

The study population consisted of 23 PTSD patients and 30 healthy subjects of both sexes (29 females, 14 males) between 18 and 56 years of age. At the first measurement point (T1), there were 23 patients and 30 controls. Both groups were matched and did not differ significantly for age [*F* (1, 51) = 3.6, *p* = 0.062], sex [*χ*^2^ (1) = 1.7, *p* = 0.192], and education level [*χ*^2^ (3) = 7.8, *p* = 0.050]. All participants gave their written informed consent. Sample characteristics are depicted in Table 1.

**Table 1:** Demographic and clinical sample characteristics.

	Full sample	Patients (PG)	Controls (CG)	*p*_*PG* vs. *CG*_
Age	32.7 (10.8)	35.8 (12.2)	30.3 (9.0)	0.075
Gender	29 females, 14 males	19 females, 4 males	20 females, 10 males	0.225
Education	43 high, 6 middle, 0 low level	15 high, 4 middle, 3 low level	28 high, 2 middle, 0 low level	0.050
BDI	11.2 (11.6)	20.7 (11.9)	4.0 (3.1)	0.000***
BSI	42.2 (44.8)	75.7 (48.9)	16.5 (14.8)	0.000***
CAPS	32.9 (32.8)	60.0 (31.9)	12.0 (11.1)	0.000***
FDS IES QMI	– – 2.0 (0.7)	0.20 (0.16) 38.9 (18.8) 2.1 (0.8)	– – 1.9 (0.6)	– – 0.298
Startle	10.8 (7.4)	11.7 (8.2)	10.1 (6.8)	0.452
ISCR	46.0 (50.0)	32.2 (32.5)	56.1 (58.0)	0.088
SCR	111.7 (119.0)	79.7 (81.5)	135.1 (136.8)	0.097
SCL	6.6 (2.2)	6.0 (1.9)	7.1 (2.3)	0.112
HR acceleration	8.5 (6.1)	8.5 (4.8)	8.6 (7.0)	0.936
HR deceleration	7.5 (3.7)	6.8 (4.0)	8.1 (3.4)	0.553
HR overall	1.0 (7.0)	1.7 (4.3)	0.5 (8.7)	0.553

PTSD patients and healthy individuals did not differ significantly in age, gender, education level, mental imagination ability (QMI-Score), and physiological base parameters (raw startle magnitude in μV, ISCR, SCR, and SCL as arbitrary data), but in the scores of the screening scales. ISCR, integrated skin conductance response (arbitrary values/10^3^), SCR, skin conductance response (arbitrary values), SCL, skin conductance level (arbitrary values/10^3^), BDI, Becks Depression Inventory, BSI, Brief Symptom Inventory, and CAPS, Clinician Administered PTSD-Scale. Values are means and standard deviations or count. Group differences in age, physiological base parameters, and the screening scales are tested via univariate ANOVAs (two-tailed). Group differences in gender and education level are tested by chi^2^-test (two-tailed). * *p* < 0.05; ** *p* < 0.01; *** *p* < 0.001.

#### PTSD patients

The PTSD patients were recruited from inpatient and outpatient therapy programs of the Department of Psychiatry and Psychotherapy, Justus-Liebig University Giessen. The inclusion criterion was posttraumatic stress disorder (ICD-10: F43.1) as the primary diagnosis. The mean duration of posttraumatic stress disorder was 5 years (± 8 years). There were 15 patients with a history of polytraumatization and 8 patients with monotraumatization. Thirteen patients showed clinically relevant dissociative symptoms (FDS > 0.13) and fifteen patients depressive symptoms (BDI > 13), which was accepted due to the well-known high overlap of depressive and PTSD symptoms (e.g., Gros et al., 2012; Stingl et al., 2020). Four patients had already participated in EMDR therapy. Ethical approval was obtained of the ethical committee of University Medicine Giessen.

#### Control subjects

Recruitment methods for the control subjects included advertisement in newspapers, newsletters, and postings on the university campus. Inclusion criterion was the absence of any psychiatric diagnosis described in the ICD-10. Two control subjects were excluded due to clinical PTSD and depression symptoms (BDI > 19, CAPS >39). Three controls and one patient had to be excluded because of low mental imagination ability (QMI score > 3.5).

#### Exclusion criteria

Individuals with documented severe mental disorders (drug abuse, dementia, schizophrenic psychosis, and intellectual disability retardation), neurological diseases (such as seizures in the anamnesis), severe hearing or visual disabilities, medication with influence on startle reflex response (benzodiazepines, buspirone, opioids), recent medication switchover within the last 2 weeks, or insufficient knowledge of German were excluded from the study. All psychiatric diagnoses were based on ICD-10 criteria and established by experienced clinical raters. Each subject completed the following screening questionnaires: Beck Depression Inventory (BDI-II), Brief Symptom Inventory (BSI), the German version of the Dissociative Experiences Scale (‘Fragebogen zu Dissoziativen Symptome’, FDS), Impact of Event Scale (IES), and Questionnaire of Mental Imagery (QMI). Demographics and clinical characteristics of the overall sample are summarized in Table 1.

### Emotion induction

Emotion induction was achieved by means of 36 imagination scripts: 12 negative, 12 neutral, and 12 positive. Negative and positive scripts were matched for their degree of arousal based on the scales of the Self-Assessment Manikin (SAM). For each stimulation condition, the same number of scripts with matched valence and arousal values was used. The scripts were largely acquired from the Affective Norms for English Text (ANET) database (Bradley and Lang, 2007b) and adapted for our German-speaking study population. Due to an insufficient number of neutral scripts in the ANET database, three scripts from Bausch et al. (2011) were added. Two further scripts were created by the study authors. It was ensured that these scripts did not differ from the ANET material in terms of relevant features (such as degree of valence and arousal, readability, text length, and situation type).

### Stimulation mode

To avoid confusion with the eyelid reflex measurement, stimulation was conducted tactilely using the Deluxe Tac/Audioscan Device Revision 5.1 from NeuroTek Corporation via rhythmically changing vibration signals applied to the person’s palms (Contact Neurotek Corporation; Wheat Ridge, CO). This instrument enabled the researcher to minimize experimenter effects by choosing a fixed duration and frequency of the vibration signals. Stimulation began at the beginning of the reading phase and stopped at the end of the imagining phase. In order to record changes in physiological baseline parameters, two thirds of the ITIs were also stimulated. ‘Bilateral stimulation’ (the right and the left hand were bilaterally stimulated in fast alternation) was compared with two control conditions: ‘monolateral stimulation’ (either the right or the left hand was stimulated) and ‘no stimulation’. Half of the monolateral stimuli were applied to the right hand and the other half to the left hand. Bilateral and monolateral stimulation differed only in the bilateral-alternating character, but not in the duration and frequency of the vibration signals.

### Self-assessment manikin

Subjective emotional responses to the script category x stimulation type conditions were rated on nine-point scales based on the SAM of Bradley and Lang (1994) immediately after each imagination trial. The subjects were instructed to think back and remember how they felt during the imagination task. Valence (from 1: negative, to 9: positive) and arousal (from 1: low arousal, to 9: high arousal) of the emotional reactions were measured.

### Startle reflex

The noise-induced startle reflex is an involuntary blink reflex that varies depending on emotional valence. The Lab Linc V Tower by Coulbourn Instruments was used to record the physiological reactions and to generate the startle tone (Contact Coulbourn Instruments; Holliston, MA). The lid closure component of the startle reflex was measured as electromyogram (EMG) of the left orbicularis oculi muscle using Ag/AgCl miniature electrodes. A 95 dB tone with a continuous white noise of 50 milliseconds was generated by the V85-04 Audio Source Module and presented binaurally via headphones. Raw EMG signal was registered by the Isolated Bioamplifier with Bandpass Filter Model V75-04. The integrated EMG signal was digitally evaluated for magnitude and latency to the peak using the Human Startle Reflex System HMS 500 Software from Coulbourn Instruments. ‘EMG magnitude’ was defined as the difference between peak EMG (highest EMG value within 20 to 150 milliseconds after the noise) and baseline EMG (EMG value within the last 100 milliseconds before) consistent with Blumenthal et al. (2005). Trials that showed a lack of reflex response, an EMG magnitude <0.1 μV, or latency to the peak >150 milliseconds were categorized as non-responses and set to 0 μV. Trials with latency to the peak <20 milliseconds, movement artifacts or excessive baseline activity were considered as missings. If a participant’s zero responses or missings exceed one third of all recorded trials, this participant was considered as a non-responder.

### Electrodermal activity

Electrodermal activity (EDA) was derived via Ag/AgCl standard electrodes on the hypothenar muscle of the non-dominant hand. The signal was recorded by the V-Amp 16 Amplifier from Brain Products GmbH with a time constant of 5 s and a voltage across the electrodes of 0.5 V (Contact Brain Products GmbH, Gilching, Germany). Arbitrary raw data were processed by the Ledalab Software (Benedek and Kaernbach, 2010). The EDA slope was recorded digitally by SCR magnitude, integrated SCR magnitude, and SCR latency to the peak. ‘SCR magnitude’ was defined as the difference between peak SCR (the highest SCR value within 900 to 4,000 milliseconds after trial onset) and baseline SCR (mean SCR value in the 1,000 milliseconds before). ‘Integrated SCR magnitude’ was calculated by the time integral of the SCR magnitude within the response window. Integrated SCR magnitude as the variable with the biggest variance was selected for analysis. Responses with latency to the peak <900 milliseconds, movement artifacts or excessive baseline activity were defined as missings. Trials with latency to the peak >4,000 milliseconds were categorized as non-responses and set to 0 μV. Participants with electrodermal nonresponding (i.e., maximum SCR magnitude equal to zero) were excluded from further analysis.

## Procedure

The study consisted of a preparation step and a measurement.

### Preparation

All volunteers gave their informed consent. Demographic data were recollected, and the screening questionnaires listed in Table 2 were completed. Subjects who did not meet the inclusion criteria did not participate in the further steps.

**Table 2:** VAS ratings before and after the experiment.

		Full Sample		Patients		Controls		PG vs. CG		*M*	*SD*	*M*	*SD*	*M*	*SD*	*p*
Pre-test		Mood	32.3	20.9	41.3	22.7	25.4	16.8	0.005**
		Arousal	34.9	20.8	40.4	24.2	26.3	24.7	0.044*
Post-test		Mood	32.4	25.3	43.1	22.5	28.6	17.3	0.011*
		Arousal	28.8	29.1	40.1	34.3	20.1	21.2	0.019*
Post vs. Pre		Mood	2.6	14.5	1.8	18.1	3.3	11.3	0.724
		Arousal	−3.6	19.8	−0.3	23.2	−6.2	16.6	0.280

Current mood and arousal before and after the experiment in both groups were assessed via Visual Analogue Scales: VAS Mood (from 1: positive, to 100: negative), VAS Arousal (from 1: low arousal, to 100: high arousal). Group comparisons were done per independent t-tests (two-tailed). Pre-post differences were tested via paired t-tests. Values are means (M) and standard deviations (SD). *p < 0.05; **p < 0.01; ***p < 0.001.

### Measurement

Directly before testing, mood (ranging from 1: positive, to 100: negative) and arousal (ranging from 1: low arousal, to 100: high arousal) were assessed via Visual Analogue Scales (VAS). Each participant was placed in front of a computer screen and electrodes and vibration pads were attached. After this preparation, script presentation and psychophysiological recordings began.

**Figure 1:**
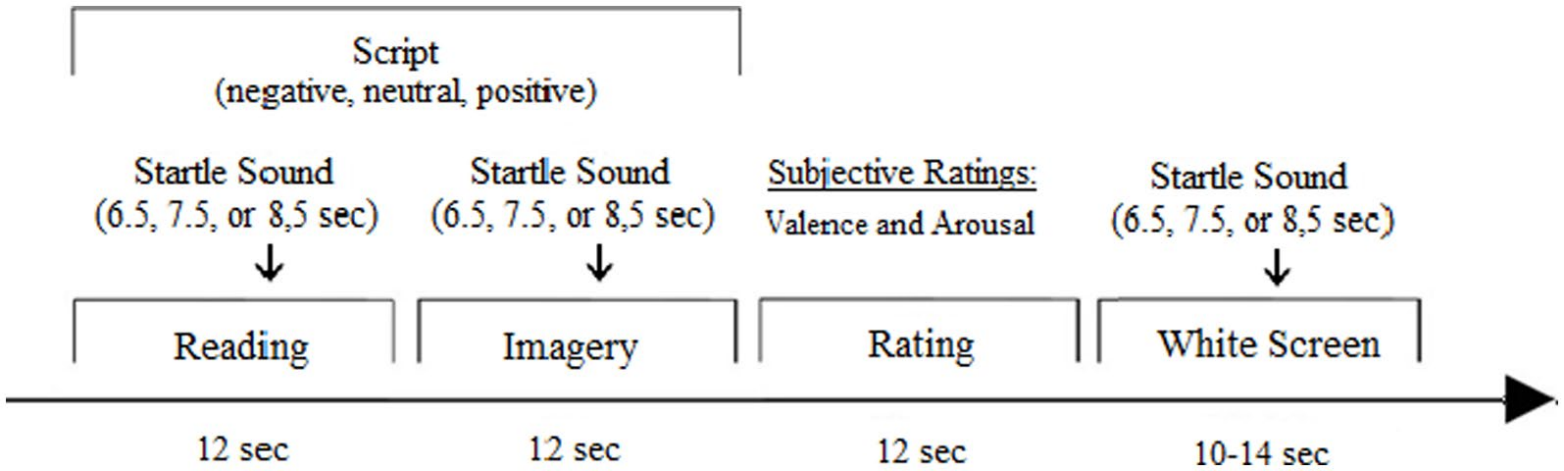
Trial sequence.

At the beginning of the measurement period, three startle reflex-triggering sounds were presented in order to reduce the potential effects of habituation. The scripts appeared in six balanced blocks in a pseudo-randomized order, interrupted by five-minute breaks. Each script consisted of the trial sequence illustrated in Figure 1: Reading (12 s), imagining (12 s), and rating the script (12 s), followed by a break in which only a white screen appeared (10–12 s). Seventy-five percent of the scripts of each category and 25 % of the white screens were combined with startle sounds. To prevent anticipation effects, the sounds appeared at variable times (6.5, 7.5, and 8.5 s after the beginning of the trials). The 10 to 12-s interval between the offset of trial n and onset of trial n + 1 was defined as the intertrial interval (ITI).

## Data reduction and analysis

The collected data were statistically processed using IBM SPSS Statistics 22.0 Software. The raw data of each subject were averaged per script category and stimulation condition. To avoid confounding with the startle noise, EDA data were analyzed only for non-startle trials. Normal distribution requirement was checked using the Kolmogorov–Smirnov test. Physiological data were square root transformed due to positive skewness. Before this step, the constant 1 was added to avoid negative values. Comparisons in VAS values were done per univariate analyses of variance (ANOVA) (two-tailed).

Hypotheses were tested using ANOVAs for repeated measures. ‘Stimulation type’ (bilateral, monolateral, no stimulation), and ‘script category’ (negative, neutral, positive) were used as within-group factors, ‘group’ (patients vs. controls) as between-group factor, and SAM valence value, SAM arousal value, startle reflex magnitude, and integrated skin conductance response as dependent factors. For all analyses, *p*-values <0.05 (two-sided) were considered statistically significant. In cases where Bonferroni corrections for multiple measurements were necessary, the calculated p-value was multiplied by the number of measurements. In cases in which sphericity could not be assumed, the Greenhouse Geisser correction for degrees of freedom was used. For the empirically well-founded hypotheses on general emotional reactivity, contrast analyses were performed. In all other cases, post-hoc tests were calculated where needed.

A post-hoc power analysis was conducted using G*Power3 (Faul et al., 2007) to compute the achieved power for the repeated measures ANOVA with two independent groups using a low effect size (partial *η*^2^ = 0.1), and an α of 0.05. Results showed that with the total sample of 53 participants the achieved power is 0.99.

## Results

### Physiological basis parameters

To exclude group differences in physiological basis parameters, ANOVAs for physiological responses during the intertrial intervals were conducted with group as between-group variable. No group differences in startle magnitude [*F*(1, 51) = 1.1, *p* = 0.296] and SCR magnitude [*F*(1, 50) < 1, *p* = 0.392] were found.

### Manipulation check

**Figure 2:**
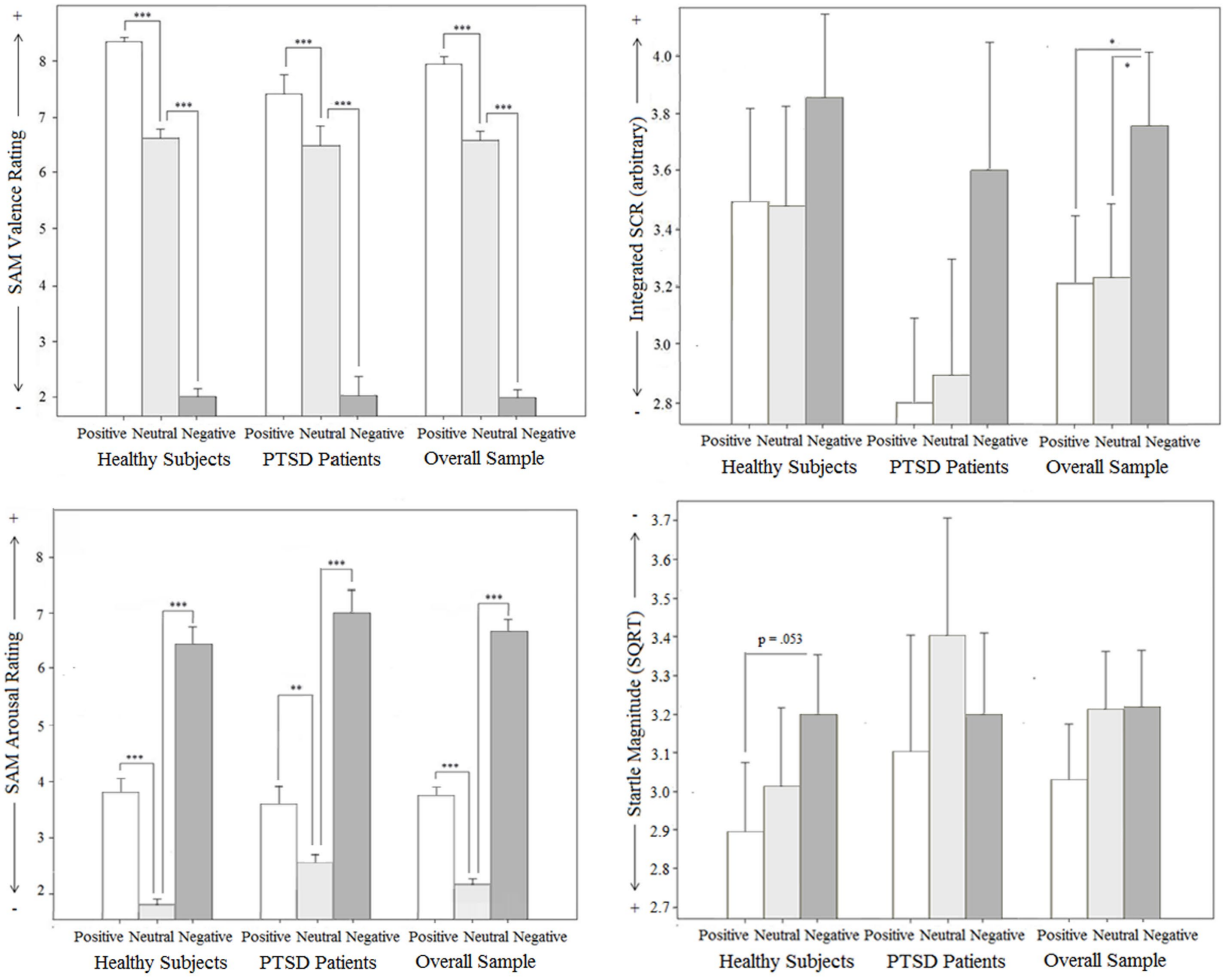
Manipulation check: Subjective (Subjective Assessment Manikin SAM for valence and arousal) and objective (Skin conductance response SCR and Startle reflex magnitude) effects for groups (PTSD and controls), total sample related to different emotional script qualities in intertrial intervals without stimulation.

For the manipulation check, a two-factorial ANOVA design with ‘script category’ (negative vs. neutral vs. positive) as a within-factor and ‘group’ (patients vs. controls) as a between-factor was used. Only trials without stimulation were included. The results showed significant valence and arousal changes during the confrontation with the different scripts in both groups, as summarized in Figure 2.

**Figure 3:**
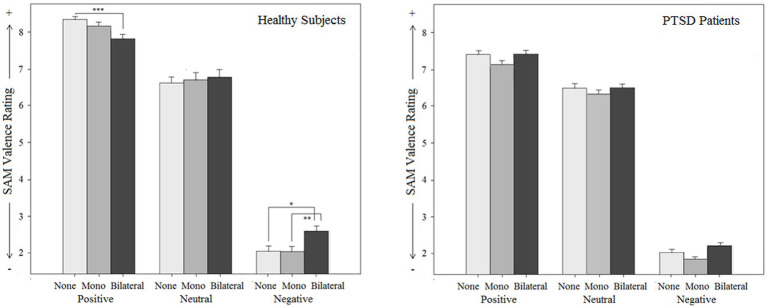
Manipulation check: Subjective (Subjective Assessment Manikin SAM for valence and arousal) and objective (Skin conductance response SCR and Startle reflex magnitude) effects for groups (PTSD and controls), total sample related to different emotional script qualities in intertrial intervals without stimulation.

#### SAM valence score

For subjective mood (SAM valence score, reaching from 1, unpleasant, to 9, pleasant), a significant main effect for ‘script category’ was found (*p* < 0.001): Positive scripts were assessed significantly more pleasant (*p* < 0.001) and negative scripts significantly more unpleasant (*p* < 0.001) than neutral scripts in both groups. The main effect for ‘group’ was not significant (*p* = 0.099), but a significant ‘script category’ x ‘group’ interaction effect was observed (*p* = 0.029). Separate ANOVAs, corrected for multiple testing, indicated that both groups differed significantly in their assessment of the positive scripts [*F*(1, 51) = 11.7, *p_c_* = 0.003], but not in their assessment of the negative [*F*(1, 51) < 1, *p_c_* = 1.000] and neutral scripts [*F*(1, 51) < 1, *p_c_* = 1.000]. For the positive scripts, a significantly lower SAM valence score (indicating less pleasance) in the patients compared to the controls was found. Interestingly, if including BDI as a covariate, this group difference was not anymore significant [*F*(1, 50) = 2.6, *p_c_* = 0.342]. Pretest arousal (VAS scale) did not have this effect [*F*(1, 50) = 7.6, *p_c_* = 0.048]. Comorbidity with depressive disorders, but not group differences in pretest arousal, thus seems to have an influence on the processing of the pleasant scripts.

#### SAM arousal score

The analysis of the SAM arousal score also showed a significant main effect for ‘script category’ (*p* < 0.001): Negative scripts and positive scripts were rated significantly more arousal-provoking than neutral ones in both groups (*p* < 0.001), as expected, but the arousal increase of the negative scripts was significantly higher (*p* < 0.001). This finding was comparable in both groups, i.e., no significant ‘script category’ x ‘group’ interaction effect (*p* = 0.116) and no main effect for ‘group’ (*p* = 0.214) were found.

#### Startle reflex magnitude

For the startle magnitude, a linear increase from positive scripts over neutral to negative scripts was expected, as shown in many studies (Lang, 1995). This finding could not be replicated: In the overall sample, the main effect for ‘script category’ was not significant (*p* = 0.074). No significant main effect for ‘group’ (*p* = 0.665) and no significant ‘script category’ x ‘group’ interaction effect were found (*p* = 0.371). Separate analyses for each group, however, indicated that this problem was restricted to the patient group: For this group, no significant main effect for ‘script category’ was observed (p_c_ = 0.766). For healthy controls, in contrast, the main effect for script category was significant (p_c_ = 0.046): Positive scripts induced a lower startle magnitude than negative scripts (*p* = 0.053). Probably because of the low sample size, this *post hoc* test did not reach the level of significance.

#### Skin conductance response

Analysis of the SCR data revealed no ‘script category’ x ‘group’ interaction effect (*p* = 0.508) and no main effect for ‘group’ (*p* = 0.280), but a significant main effect for ‘script category’(*p* = 0.005): Negative scripts induced a significantly larger SCR than neutral scripts (p = 0.005) and positive scripts (*p* = 0.035) in the overall sample. For positive scripts, no significant SCR increase compared to neutral scripts was found (*p* = 1.000). This finding is in contrast to previous studies which reported comparable SCR increase for positive and negative scripts compared to neutral scripts. The positive scripts in the present study were thus not arousal-provoking enough. To provoke a higher arousal also for the positive affective stimuli, personalizing the scripts might be helpful. Another possibility might be using pictures instead of scripts.

Separate analyses for both groups, corrected for multiple testing, indicated that in the patient group the main effect for script category stayed significant (*p* = 0.032), whereas in the control group it did not (*p* = 0.506). That is the patients showed a stronger physiological arousal increase during the negative scripts.

To sum, while the patients did not differ from the controls in physiological basis variables, the affect-related modulation of the startle reflex magnitude was significantly disturbed with a missing startle inhibition for positive scripts. This finding correlated with significantly less subjective pleasance in the patients during imagining the positive scripts and a significantly worse mood (measured via VAS scales) at the beginning and the end of the experiment. These particularities might indicate a general dysfunction of the behavioral approach system resulting from the psychopathological characteristics of this sample. To explore this, a covariance analysis was conducted with SAM valence rating of the positive scripts as a dependent variable, ‘group’ as an independent variable, and PTSD specific psychopathological covariates (CASP, IES, BSI). When holding these factors constant, the group difference in the SAM valence score was no longer significant [CASP: *F*(1,50) = 3.9, *p* = 0.055; IES: *F*(1, 50) = 3.0, *p* = 0.088] or at least marginally reduced [BSI: *F*(1, 50) = 4.1, *p* = 0.048, compared to a *p* = 0.12]. Depression specific covariates (BDI) also eliminated the group difference [*F*(1, 50) = 2.6, *p* = 0.114], but not situational factors such as mood [VAS mood: *F*(1, 50) = 7.2, *p* = 0.010] and arousal [VAS arousal: *F*(1, 50) = 7.6, *p* = 0.008] before the experiment or the imagination ability of the subjects [QMI: *F*(1, 50) = 12.0, *p* = 0.001]. For these reasons, PTSD-typical maladaptive emotion processing processes as well as comorbidity with depressive symptoms can be used as an explanation.

### Stimulation effect

**Figure 4:**
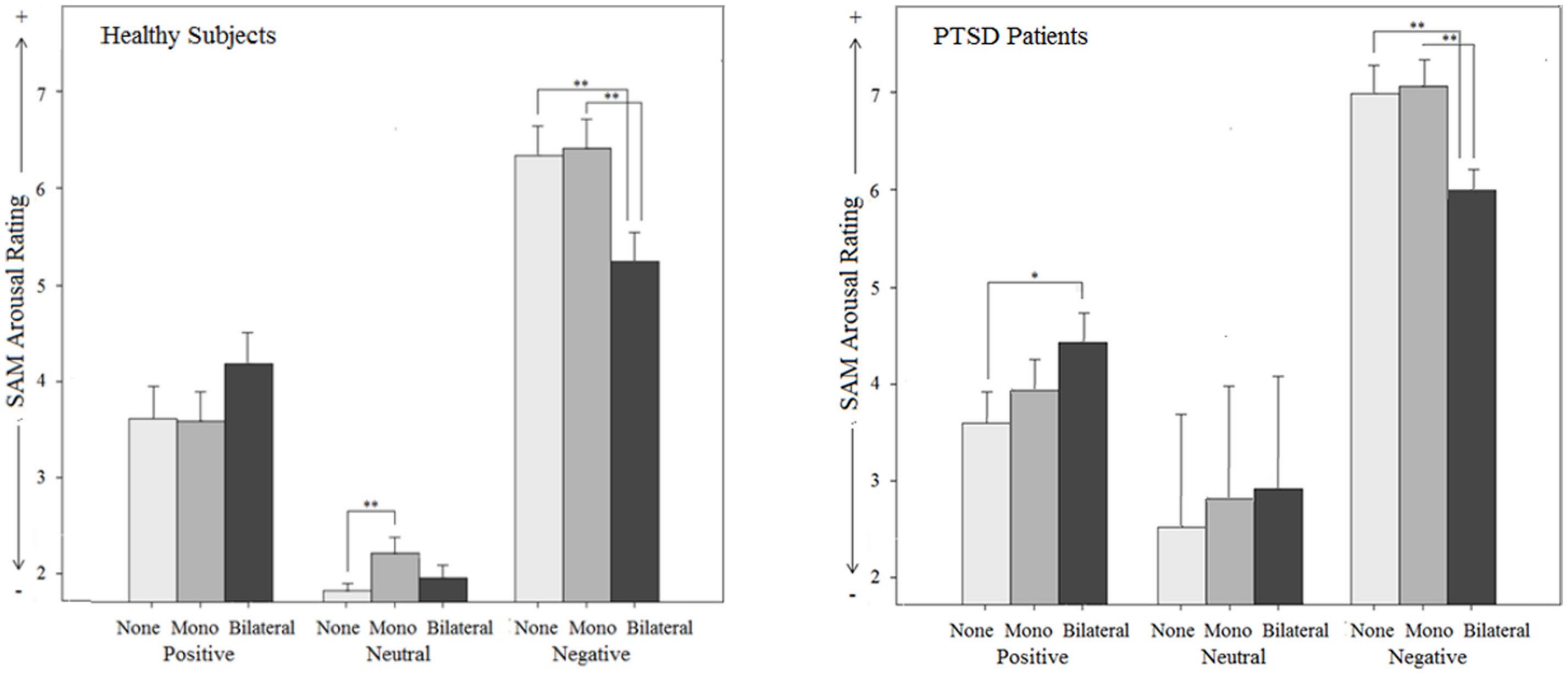
Subjective effects on arousal (Subjective Assessment Manikin SAM) by different types of stimulation (mono-, bilateral, no stimulation) for both groups (PTSD and controls) in relation to different emotional script qualities.

**Figure 5:**
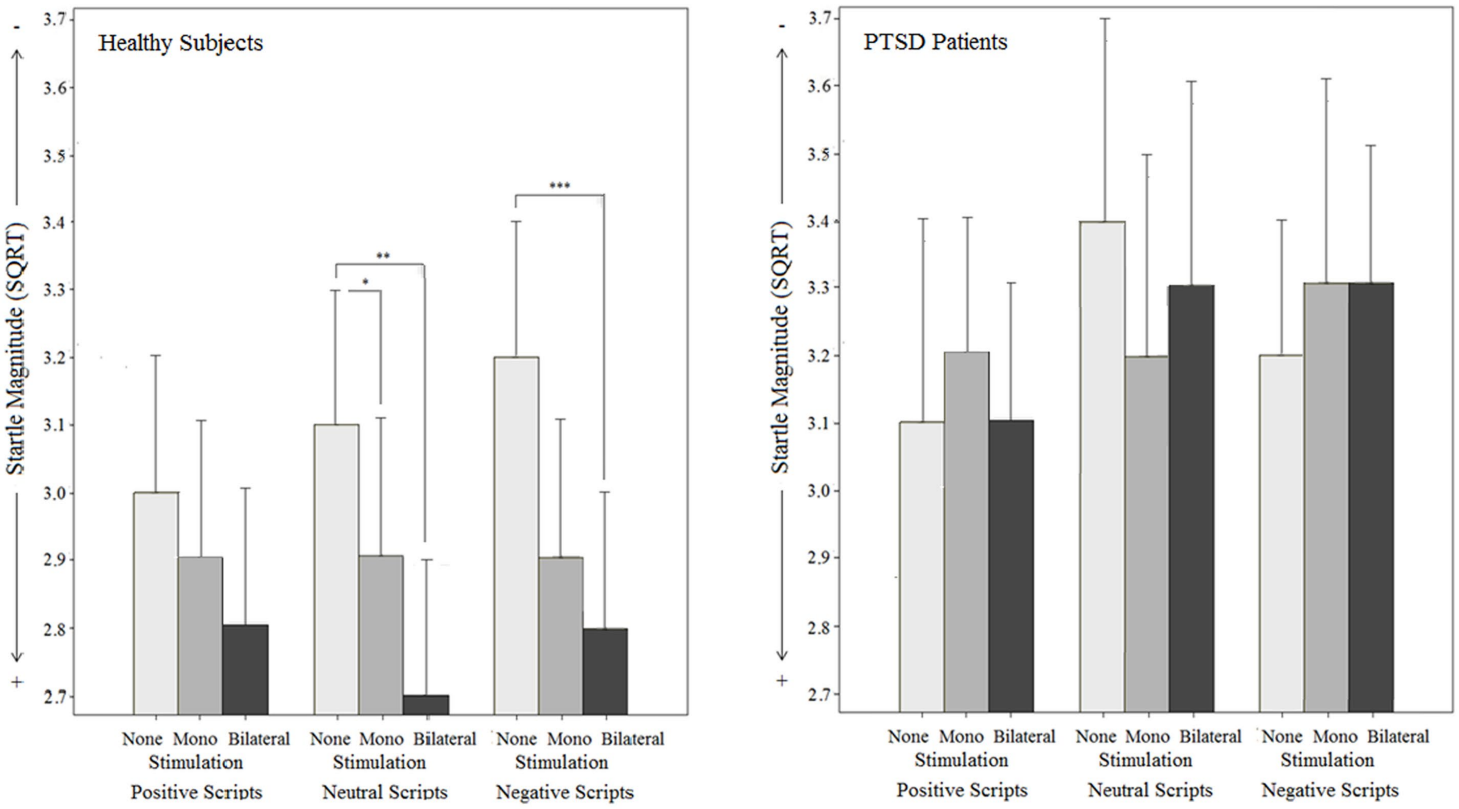
Objective effects on Startle reflex magnitude by different types of stimulation (mono-, bilateral, no stimulation) for both groups (PTSD and controls) in relation to different emotional script qualities.

**Figure 6:**
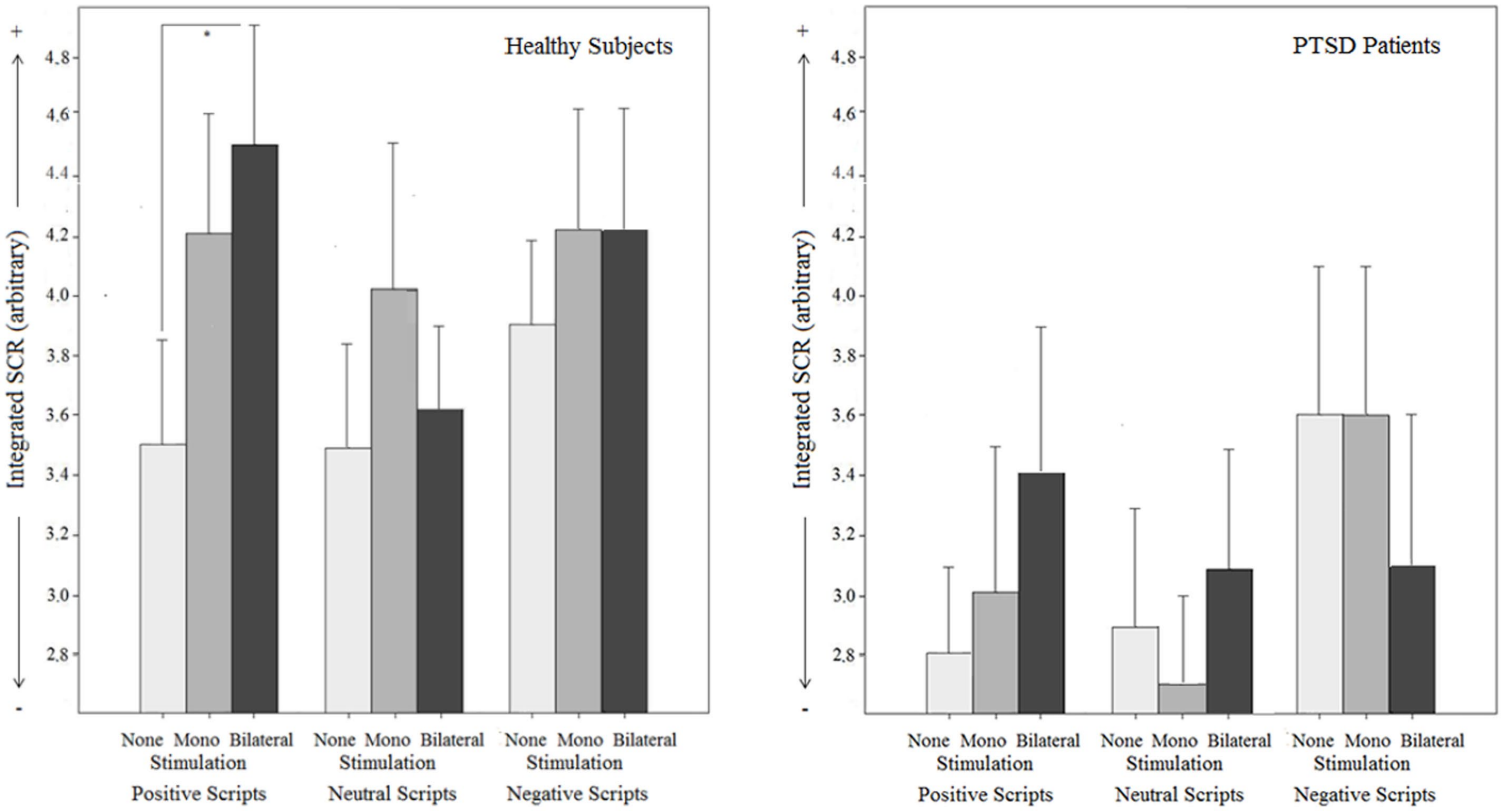
Objective effects on Skin conductance response (SCR) by different types of stimulation (mono-, bilateral, no stimulation) for both groups (PTSD and controls) in relation to different emotional script qualities.

Stimulation effect was examined using a three-factorial ANOVA design with ‘script category’ (negative vs. neutral vs. positive) and ‘stimulation type’ (bilateral vs. monoliteral vs. none stimulation) as within-factors and ‘group’ (patients vs. controls) as a between-factor. The results are depicted in Figures 2–6.

**Figure 7:**
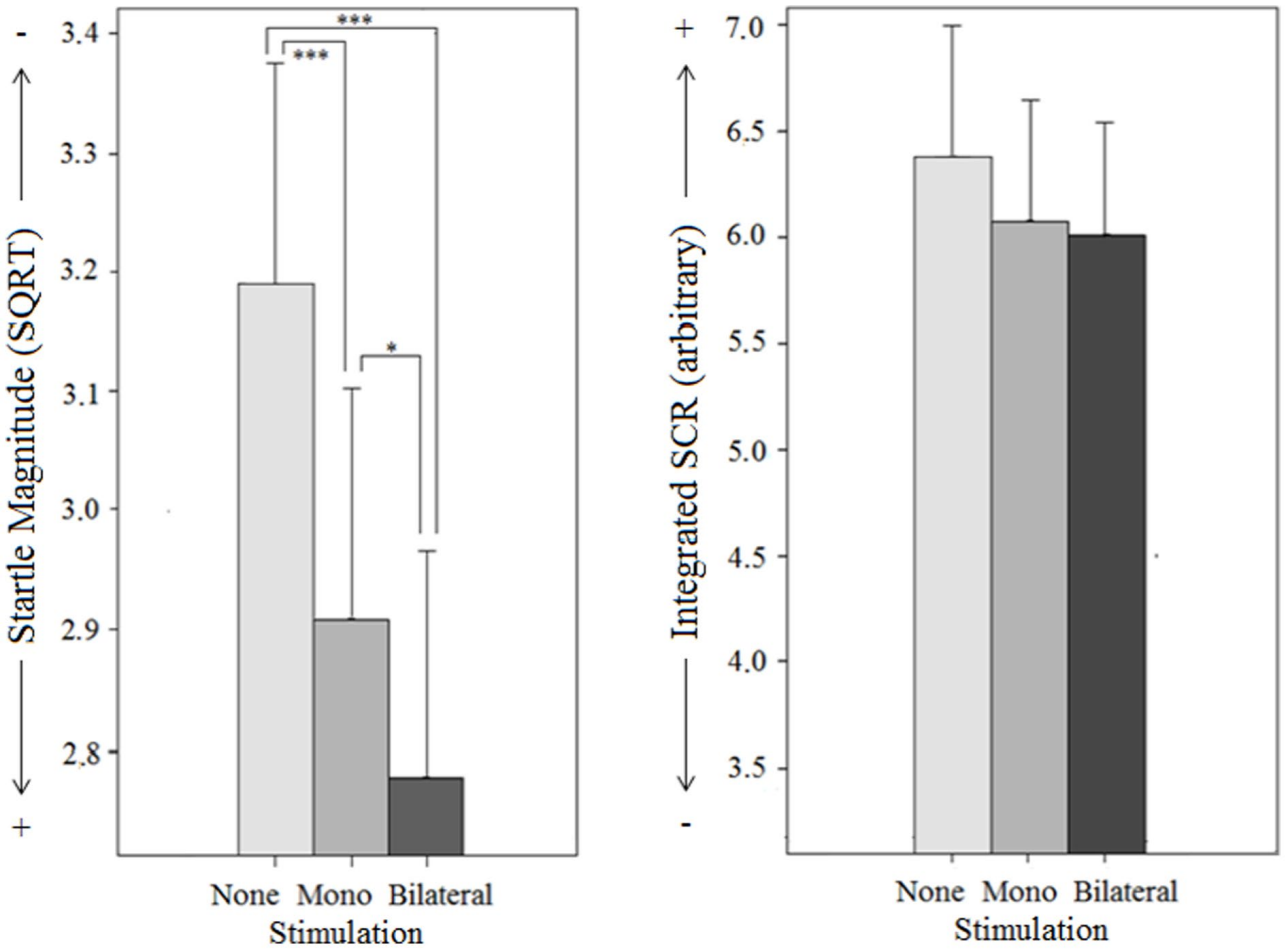
Objective effects on Startle reflex magnitude and Skin conductance response (SCR) by different types of stimulation (mono-, bilateral, no stimulation) for both groups (PTSD and controls) in intertrial intervals without emotion induction.

#### SAM valence score

The findings for the SAM valence score (reaching from 1 = unpleasant, to 9 = pleasant) is first reported for the overall sample: A significant main effect for script category (*p* < 0.001), a main effect for stimulation type (*p* = 0.031), and a stimulation type x script category interaction effect was found (*p* = 0.019). *Post hoc* tests indicated that bilateral stimulation significantly decreased negative feelings while viewing *negative scripts* (*p* = 0.032), whereas monolateral stimulation did not (*p* = 1.000). For *positive scripts*, a significant decrease in positive feelings was observed under bilateral stimulation (*p* = 0.004), but not under monolateral stimulation (*p* = 0.170) Bilateral stimulation thus reduced subjective emotion intensity in the expected direction. For *neutral scripts*, there were no changes in emotional valence neither for bilateral (*p* = 1.000) nor for monolateral stimulation (*p* = 1.000).

The stimulation type x group interaction effect (*p* = 0.353) and the script category x stimulation effect x group interaction effect were not significant (*p* = 0.133). However, separate ANOVAs for each group (corrected for multiple testing) indicated that the valence-modulating effect of the stimulation was specific for healthy individuals, where a significant script category (positive vs. neutral vs. negative) x stimulation type (bilateral vs. monolateral vs. no stimulation) interaction effect was observed (*p*_c_ = 0.002). In PTSD patients, this effect did not reach the level of significance (*p*_c_ = 1.000).

As a side result, a significant main effect for group [*F*(1, 51) = 4.9, *p* = 0.032] was found: SAM valence score was significantly lower in patients compared to controls, indicating that the patients generally felt more negative while imagining the scripts.

#### SAM arousal score

For the SAM arousal score (reaching from 1, low arousal, to 9, high arousal), a significant script category x stimulation type interaction effect (*p* < 0.001) was observed in the overall sample: *Post hoc* tests showed that bilateral stimulation decreased subjective arousal exclusively for negative scripts (MD_bi vs. none_ = −1.11, *p* = 0.000, 95% CI [−1.64,-0.58]). For positive scripts, a reversed effect was detected with a significantly larger subjective arousal under bilateral stimulation compared to no stimulation [MD_bi vs. none_ = 0.58, *p* = 0.001, 95% CI (0.22, 0.94)]. The assessment of neutral scripts was not influenced [MD_bi vs. none_ = 0.19, *p* = 0.072, 95% CI (−0.01, 0.38)]. Bilateral stimulation thus decreased arousal for negative content and increased arousal for positive content. Monolateral stimulation had no effect on arousal during negative [MD_mono vs. none_ = 0.01, *p* = 1.000, 95% CI (−0.52, 0.54)] and positive scripts [MD_mono vs. none_ = −0.03, *p* = 1.000, 95% CI (−0.60, 0.53)], but increased arousal for neutral scripts [MD_mono vs. none_ = 0.30, *p* = 0.009, 95% CI (0.06,0.55)]. This interaction effect was significant both in the patient sample (*p* < 0.001) and the control sample (*p* < 0.000). The script category x stimulation type x group interaction effect was not significant (*p* = 0.559), i.e., stimulation effect did not differ between the groups. The main effect for stimulation type (*p* = 0.202), the stimulation type x group interaction effect (*p* = 0.660), and the main effect for group (*p* = 0.111) were also not significant.

#### Startle reflex magnitude

The analysis of the startle magnitude revealed a significant main effect for stimulation type (*p* = 0.008) in the overall sample, but no stimulation type x script category interaction effect (*p* = 0.515): Bilateral stimulation (MD_bi vs. none_ = −1.55, *p* = 0.013, 95% CI [−0.28,-0.27]) decreased startle magnitude compared to no stimulation independently of script category. For monolateral stimulation, no significant stimulation effect was found [MD_mono vs. none_ = −0.91, *p* = 0.269, 95% CI (−0.22, 0.04)]. The main effect for group (*p* = 0.324) and the stimulation type x script category x group interaction were not significant (*p* = 0.538), but valence-specific startle-reducing effect exclusively for the controls was found. The data further revealed a significant interaction effect between stimulation type and group (*p* = 0.010):

For *healthy individuals*, the main effect for stimulation type was significant (*p_c_* < 0.001) with a lower startle magnitude for bilateral stimulation (MD_bi vs. none_ = −0.30, *p* < 0.001, 95% CI [−0.44,-0.17]) and monolateral stimulation (MD_mono vs. none_ = −0.20, p = 0.009, 95% CI [−0.35,-0.04]) compared to no stimulation. Moreover, separate ANOVAs (manually corrected for multiple testing) indicated, that bilateral stimulation significantly reduced startle magnitude for negative (*p* < 0.001) and neutral scripts (*p* < 0.001), whereas no effect was found on positive scripts (*p* = 0.379). Monolateral stimulation had no effect on startle magnitude when the negative scripts (*p* = 0.063) and positive scripts (*p* = 1.000) were regarded separately, but a significant effect on the neutral scripts was found (*p* = 0.009).

For *PTSD patients*, the main effect for stimulation type was not significant (*p_c_* = 1.000). This was the same for all script categories, i.e., there was no stimulation type x script category interaction effect neither for healthy individuals (*p_c_* = 0.762) nor for patients with PTSD [*F*(2.7, 58.6) < 1, *p_c_* = 1.000].

#### Skin conductance response

For SCR magnitude, the script category x stimulation type interaction effect [*F*(3.4,167.8) = 2.0, *p* = 0.117] was not significant, but separate analyses for each script category (manually corrected for multiple testing) showed a differential effect: When analyzing the positive scripts, a significant SCR increase under bilateral stimulation [MD_*bi* vs. *none*_ = 2.35, p_c_ = 0.027, 95% CI (0.47, 4.23)], but not under monolateral stimulation [MD_*mono* vs. *none*_ = 1.23, p_c_ = 0.591, 95% CI (−0.39, 2.84)] compared to no stimulation was detected. For the negative scripts, neither bilateral stimulation [MD_*bi* vs. *none*_ = −0.27, *p*_c_ = 1.000, 95% CI (−1.84, 1.78)] nor monolateral stimulation [MD_*mono* vs. *none*_ = 0.40, *p*_c_ = 1.000, 95% CI (−1.05, 1.86)] had an SCR-modulating effect. Similarly, there was no SCR-modulation effect under bilateral [MD_*bi* vs. *none*_ = 0.34, *p*_c_ = 1.000, 95% CI (−1.05, 1.73)] or monolateral stimulation [MD_*mono* vs. *none*_ = 0.59, *p*_c_ = 1.000, 95% CI (−0.99, 2.17)] for the neutral scripts. These findings indicate a valence-dependent SCR-increasing effect of bilateral stimulation. No script category x stimulation type x group interaction [*F*(3.4,167.8) = 1.0, *p* = 0.399], no stimulation type x group interaction [*F*(2,100) = 1.7, *p* = 0.184], and no main effects for stimulation type [*F*(2,100) = 1.871, *p* = 0.159] and group [*F*(1, 50) = 3.1, *p* = 0.084] were found.

### Intertrial intervals

To examine stimulation influence on physiological basis parameters, ANOVAs were calculated during the intertrial intervals with ‘stimulation type’ (bilateral vs. monolateral vs. none) and ‘group’ (patients vs. controls) as independent variables. The findings are depicted in Figure 7.

For startle magnitude, no main effect for group [*F*(1, 51) = 1.1, *p* = 0.296], but a strong significant main effect for stimulation type [*F*(1.7,85.2) = 16.0, *p* < 0.001] and a significant group x stimulation type interaction effect [F(1.7, 85.2) = 5.9, *p* < 0.006] were found: In the control group, there was a significant main effect for stimulation type [*F*(2, 58) = 33.8, *p* < 0.001], i.e., startle magnitude was significantly lower under bilateral stimulation [*MD*_*bi* vs. *none*_ = −0.41, *p* < 0.001, 95% CI (−0.56, 0.27)] and monolateral stimulation [*MD*_*mono* vs. *none*_ = −0.28, *p* < 0.001; 95% CI (−0.42,-0.14)] than under no stimulation. However, the effect of bilateral stimulation was significantly stronger [*MD*_*bi* vs. *mono*_ = −0.13, *p* = 0.011, 95% CI (−0.24,-0.03)]. In the patient group, the main effect for stimulation type was not significant [*F*(2, 44) < 1, *p* = 0.385].

Integrated SCR during the ITIs did not vary following stimulation type. The main effect for stimulation type was not significant [*F*(2,100) < 1, *p* = 0.931]. This corresponds to the results of Gunter and Bodner (2008), who observed a stimulation-induced physiological arousal increase only in the presence of emotional stimuli. The main effect for group [*F*(1, 50) < 1, *p* = 0.392] and the group x stimulation type interaction effect [*F*(2, 100) = 2.3, *p* = 0.111] were not significant.

### Visual analogue scales

Before and after the complete script presentation and measurement procedure, mood (ranging from 1, positive, to 100, negative) and arousal (ranging from 1, low arousal, to 100, high arousal) were assessed via Visual Analogue Scales (VAS).

Comparisons between patients and controls (PG vs. CG) were done via univariate ANOVAs (two-tailed). Both groups differed significantly in their subjective mood and arousal (Table 2): Patients with PTSD showed significantly worse mood [*F*(1, 51) = 8.6, *p* = 0.005] and significantly higher arousal [*F*(1, 51) = 4.3, *p* = 0.044] compared to healthy subjects before the experiment. Comparable group differences for mood [*F*(1, 51) = 7.0, *p* = 0.011] and arousal [*F*(1, 51) = 6.8, *p* = 0.012] were found for the post-test rating, indicating that the emotion task was experienced as more stressful by the patients. This finding might indicate a limited ability to cope with affective stimuli in patients with PTSD.

Pre- to post-test difference values (i.e., VAS values before and after the complete script presentation and measurement procedure) were compared via univariate ANOVAs (two-tailed). Pre- to post-test difference values for mood [*F*(1, 51) < 1, *p* = 0.724] and arousal [*F*(1, 51) = 1.192, *p* = 0.280] did not significantly differ between both groups. However, separate pre-to post-test comparisons for each group indicated that arousal significantly decreased from pre to post in healthy subjects [*F*(1, 29) = 4.2, *p* = 0.049], whereas no changes in PTSD patients were found [*F*(1, 22) < 1, *p* = 0.958]. Whereas the controls thus showed a habituation effect, the patients were as stressed after measurement as at the beginning of the experiment. Mood was stable over time in both the patient [*F*(1, 22) < 1, *p* = 0.633] and the control group [*F*(1, 29) = 2.5, *p* = 0.123].

## Discussion

This study aimed to contrast PTSD patients vs. healthy volunteers in their subjective and objective reaction patterns in an emotional imagination paradigm. Additionally, the effects of bilateral stimulation as applied in EMDR therapy were investigated in both groups.

### Group differences in baseline parameters

On the subjective level, patients reported significantly higher arousal and a worse mood than healthy individuals before the testing and missing habituation to the testing situation. That finding is in line with persistent hyperarousal as one criterion of PTSD. However, on the objective measurable level, no increased SCRs during the ITIs as indicators of hyperarousal were observed. This corresponds with previous studies (Metzger et al., 1999; Orr et al., 2003), where the subjectively reported hyperarousal could not be objectively confirmed. Furthermore, patients showed objectively no hyperreactivity as no increased startle reflex response was found.

The observed discrepancy - subjective hyperarousal without a physiological correspondence - in the patients can only be discussed hypothetically here against the background of existing findings and requires further exploration. Two considerations are suggested as preliminary explanatory hypotheses: 1. Dissociation: dissociation is present in many mental disorders and disrupts mental functions (Lyssenko et al., 2018). It affects the integration of consciousness, memory, thinking, emotion, sensorimotor functions, identity, and behavior (APA, 2013). In PTSD, dissociation can serve as a psychobiological defense mechanism to cope with traumatic experiences and avoid emotional distress (Dalenberg et al., 2012). Even if there is explicitly a “dissociative subtype of PTSD” (APA, 2013; DSM-V), however, significantly more PTSD patients suffer from impaired functioning due to milder forms of dissociative phenomena which are experienced regularly, in response to ostensibly minor stressors (Fani et al., 2019). According to this, it can be hypothesized that the lack of physiological responses to emotional stimuli might be the result of autonomic blunting due to a dissociative process (Schäflein et al., 2018; Beutler et al., 2022). 2. PTSD-specific cognitive processing style: evaluating an uncertain situation and being prepared to react immediately to specific learned threats, PTSD patients are operating in a state of possibility thinking using past evidence. Therefore, all possible outcomes or circumstances must be considered in relation to their (traumatic) memory, rather than the typical probability thinking of the average individual. Instead of trusting that something probably will or will not happen, PTSD patients tend to have in mind distressing worst-case scenarios and consider them. Studies examining information processing in PTSD support such a “sense of current threat” in PTSD patients according to their overestimation of the probability of the traumatic event reoccurring (Ehlers and Clark, 2000; Regambal and Alden, 2012). The resulting constant worrying engagement could be subjectively perceived and interpreted as hyperarousal. A combination of both - subjective hyperarousal as a result of catastrophizing cognitive processes (also in respect to the testing situation) and physiological non-responding as a result of dissociation - is also possible and consistent with the findings.

### Group differences in emotional reactivity

According to the results of McDonagh-Coyle et al. (2001), patients experienced the pleasant scripts significantly less positively than healthy subjects, and the negative scripts were more aversive in their subjective judgment. However, the expected physiological effects on the startle did not occur in the PTSD patients: no startle inhibition during the pleasant scripts (non-stimulation condition) and no startle potentiation for unpleasant scripts was observed, even if SCR (i.e., attention) for the scripts was significantly increased (compared to healthy participants). These effects were correlated with the BDI-II score, but not with situational factors such as the negative pre-test mood. For this reason, a dysfunction of the behavioral approach system in the patient group for positive emotions can be assumed here, which may have been influenced by depressive comorbidity. The different reaction patterns to induced negative emotion in healthy individuals and PTSD patients contrast with a previous study where both groups showed similar reactivity to negative, non-trauma-associated stimuli (Tarrier et al., 2002).

### Group differences in the various stimulation conditions

The results are first reported for the subjective level: BLS affected the subjectively felt arousal equally in healthy individuals and patients by reducing it in both negative and positive emotion induction. The same was observed regarding valence where BLS reduced negative and positive valence ratings in both groups, whereas no such BLS effects were observed presenting neutral scripts. This should be considered when EMDR is used to install or reinforce positive resources: In accordance with our results, fast BLS could weaken the subjective arousal and valence of positive resources. However, in practice, slow BLS are often used intuitively (as they have not been further validated), as in the “EMDR Resource Development and Installation (RDI)” protocol (Korn and Leeds, 2002).

When comparing the impact of BLS at a physiological level significant differences between the two groups were found: In healthy subjects, BLS reduced startle SCR for positive scripts which can be interpreted as an increase in attention to the positive stimuli. Moreover, a significantly reduced startle reflex response in the absence of affective stimuli potentiation in negative scripts, which can be understood as physiological confirmation of a decrease in negative valence resp. aversiveness. Under BLS healthy individuals also showed an increased was observed during BLS, which represents an effect of a generally reduced affective responsiveness.

In contrast, the patients did - contrary to their subjective assessments - *not* show any corresponding physiological relaxation effects when being exposed to BLS. To interpret this finding, one could refer to the concept of dissociation and/or the above-described information processing model in PTSD: emotion induction via scripts means for patients to find themselves in a new and uncertain situation with emotional content, in which the crucial probability assessment is automatically carried out. This requires the use of higher cognitive processes, which are taxed by the dual task of BLS, in that less attentional capacity is available and therefore the subjective load is diminished.

In the monolateral stimulation variant, increased subjective arousal was found in both groups with neutral scripts, while no effect was found with the presentation of pleasant or unpleasant scripts. Monolateral stimulation had no effect on either subjective valence or physiological parameters (startle reflex, SCR).

These findings of different effects concerning the type of stimulation are consistent with applied studies that report more specific effects in BLS compared to monolateral stimulation (e.g., Stingl et al., 2022).

## Implications

This work should be understood as a basic study expanding the knowledge about commonalities and differences between PTSD patients and healthy controls related to subjective and physiological basic parameters and emotional reactivity. In addition, the differential effects of bilateral stimulation on individuals with/without PTSD were examined, where monolateral stimulation and no stimulation served as control conditions.

Applying BLS has several advantages over monolateral stimulation – it helps to reduce subjective arousal, diminishes subjective strain, and enhances positive valence when imagining scrips with emotional content. These effects correspond to the intended application of BLS in EMDR therapy where it is used to process distressing experiences and to reinforce positive mental images and emotions.

Furthermore, the described corresponding physiological effects of BLS could only be demonstrated in healthy individuals, but not in PTSD patients. Since the reasons for this could be comorbidity with depression or dissociative symptoms, which often occur in patients with PTSD, adequate treatment of both should precede or be integrated in EMDR therapy. This might include, for example, applying antidepressant treatment and/or implementing strategies for dealing with dissociation, as preceding steps to avoid attenuation of bilateral stimulation effects.

The reported results may also help to better categorize EMDR therapy within evidence-based psychotherapies. According to the framework for evidence-based psychosocial interventions by David and Montgomery (2011), EMDR has proven its effectiveness in practical application, but the underlying theory of the AIP model is still insufficiently investigated. Therefore, the EMDR therapy can be classified within the framework in category II “intervention-guided psychotherapy.” Here, the dismantling study design provides information for further validation of the underlying EMDR theory: consistent with the disease and treatment theory of PTSD, the therapeutic package of EMDR aims to improve emotion regulation skills in PTSD patients, because dysfunctional emotion regulation skills play a crucial role in the initiation and maintenance of the disorder. As predicted by EMDR theory, the application of BLS reduced subjective stress and increased positive valence, while monolateral stimulation did not. Further studies should examine the assumptions and mechanisms of change of EMDR in order to clarify the scientific level of EMDR therapy and distinguish it from pseudoscientific psychotherapy. Common factors (see Wampold, 2001) that might additionally contribute to therapeutic change should also be considered when investigating the effects of the EMDR therapeutic package.

## Limitations

The study was conducted with a relatively small sample size, and some of the effects found in this sample were small. There was no pre-registration, but the authors will provide insight into the raw data upon request. The dismantling design was conducted as a laboratory study with a standardized procedure using a block-wise standardized emotion induction, which is an approximation of a true EMDR therapy. Incidentally, such a direct comparison cannot be performed in the context of a real clinical EMDR study, since a comparable trauma confrontation in healthy subjects is not possible. Regarding the emotion induction used, the positive scripts were less arousing compared with the negative ones, which may have influenced the inhibition of the startle reflex. This problem, also observed in previous studies (Cuthbert et al., 1990; Vanoyen Witvliet and Vrana, 1995; Miller et al., 2002), could be addressed by personalizing the scripts. Stimulation was performed tactilely, in contrast to clinical practice in which horizontal eye movements are preferred. Even though studies for tactile and visual stimulation show comparable effects, the results of this work cannot automatically be transferred to other stimulation types. Another possible limiting factor is the duration of stimulation. In EMDR therapy, stimulation lasts longer, and the end point of stimulation before switching to a new association pathway depends on the valence of the very last memory that emerged. The current stimulation is stopped and a new association pathway is processed only when a neutral or positive memory emerges. This change in emotional valence is often indicated by subtle changes in facial expression or body posture, which the therapist uses for guidance. Stopping the stimulation too quickly without taking these markers into account may result in a lack of the arousal- and valence-modulating effects of the stimulation. For this reason, the stimulation phases should be extended and individualized in future studies. Since the majority of the sample consisted of participants who had not previously been treated with EMDR, the influence of previous EMDR experience cannot be determined here, nor can the question of how differences between (usually more severely disturbed) inpatients and outpatients might affect the results. An important question for future studies is whether the observed stimulation effects are stable over time. For this reason, a follow-up measurement would be helpful. Although previous studies showed no significant differences between tactile and non-tactile stimulation (Nieuwenhuis et al., 2013), the present results are so far limited to the tactile stimulation type.

## Conclusion

In healthy individuals, bilateral stimulation had a significant influence on emotional reactivity both in subjective and physiological terms: Startle reflex response during imaging negative scripts was reduced, and SCR (i.e., attention) for positive scripts was increased. Monolateral stimulation did not have a comparable effect. These findings are promising, as bilateral stimulation is used for weakening negative (e.g., trauma-related) imaginal scenes and for confirming positive imaginal scenes during EMDR therapy (Shapiro, 2017). For PTSD patients, however, only a subjective arousal-reducing effect, but no concomitant physiological changes were found. This could have resulted from depressive comorbidity, dissociation, or cognitive processing style in connection with the comparably low duration of the stimulation.

Overall the study showed that PTSD patients and healthy subjects were *not* comparable in their subjective (psychological) and objective (physiological) baseline and response patterns to BLS in the emotional imagination paradigm, so the effects observed in one group cannot simply be generalized to the other.

## Data availability statement

The raw data supporting the conclusions of this article will be made available by the authors, without undue reservation.

## Ethics statement

The studies involving humans were approved by the Ethics committe of the faculty of medicine uniyersity of Giessen. The studies were conducted in accordance with the local legislation and institutional requirements. The participants provided their written informed consent to participate in this study.

## Author contributions

VP: Conceptualization, Data curation, Formal analysis, Investigation, Methodology, Project administration, Resources, Software, Supervision, Validation, Visualization, Writing – original draft, Writing – review & editing. GS: Data curation, Methodology, Writing – review & editing. BH: Writing – review & editing. ES: Writing – review & editing. FR: Writing – review & editing, Methodology. MS: Conceptualization, Data curation, Formal analysis, Investigation, Methodology, Resources, Software, Supervision, Validation, Visualization, Writing – original draft, Writing – review & editing.
